# Body composition and risk factors for cardiovascular disease in global multi-ethnic populations

**DOI:** 10.1038/s41366-023-01339-9

**Published:** 2023-07-17

**Authors:** Jennifer L. Carter, Noraidatulakma Abdullah, Fiona Bragg, Nor Azian Abdul Murad, Hannah Taylor, Chin Siok Fong, Benjamin Lacey, Paul Sherliker, Fredrik Karpe, Norlaila Mustafa, Sarah Lewington, Rahman Jamal

**Affiliations:** 1 Clinical Trial Service Unit and Epidemiological Studies, Nuffield Department of Population Health, University of Oxford, Richard Doll Building, Old Road Campus, Oxford, OX3 7LF UK; 2 UKM Medical Molecular Biology Institute (UMBI), Jalan Yaacob Latiff, 56000 Cheras, Kuala Lumpur, Malaysia; 3 Medical Research Council, Population Health Research Unit, University of Oxford, Oxford, UK; 4 Oxford Centre for Diabetes, Endocrinology and Metabolism, University of Oxford, Churchill Hospital, Headington, OX3 7LE UK; 5 Department of Medicine, Faculty of Medicine, University Kebangsaan Malaysia, 56000 Cheras, Kuala Lumpur, Malaysia

**Keywords:** Epidemiology, Risk factors, Cardiovascular diseases

## Abstract

**Background:**

No large-scale studies have compared associations between body composition and cardiovascular risk factors across multi-ethnic populations.

**Methods:**

Population-based surveys included 30,721 Malay, 10,865 Indian and 25,296 Chinese adults from The Malaysian Cohort, and 413,737 White adults from UK Biobank. Sex-specific linear regression models estimated associations of anthropometry and body composition (body mass index [BMI], waist circumference [WC], fat mass, appendicular lean mass) with systolic blood pressure (SBP), low-density lipoprotein cholesterol (LDL-C), triglycerides and HbA1c.

**Results:**

Compared to Malay and Indian participants, Chinese adults had lower BMI and fat mass while White participants were taller with more appendicular lean mass. For BMI and fat mass, positive associations with SBP and HbA1c were strongest among the Chinese and Malay and weaker in White participants. Associations with triglycerides were considerably weaker in those of Indian ethnicity (eg 0.09 [0.02] mmol/L per 5 kg/m^2^ BMI in men, vs 0.38 [0.02] in Chinese). For appendicular lean mass, there were weak associations among men; but stronger positive associations with SBP, triglycerides, and HbA1c, and inverse associations with LDL-C, among Malay and Indian women. Associations between WC and risk factors were generally strongest in Chinese and weakest in Indian ethnicities, although this pattern was reversed for HbA1c.

**Conclusion:**

There were distinct patterns of adiposity and body composition and cardiovascular risk factors across ethnic groups. We need to better understand the mechanisms relating body composition with cardiovascular risk to attenuate the increasing global burden of obesity-related disease.

## Introduction

The global burden of obesity-related disease has been increasing over the last three decades, with over two-thirds of deaths due to cardiovascular disease [1]. However, metabolic risks associated with adiposity differ between populations, and these differences are not completely understood. In the large population-based Malaysian Cohort (TMC) study, Malay and Indian groups reported equivalent levels of obesity (indicated by body-mass index [BMI]), but Indians reported a much higher prevalence of type II diabetes mellitus (28% vs 19%) alongside less dyslipidaemia (42% vs 51%) [2]. One of the only studies large enough to reliably examine prospective associations with vascular disease in South Asians showed little association between BMI and vascular mortality, contrasting the strong positive associations with obesity observed in European and North American populations [3, 4]. This finding was despite BMI being strongly positively correlated with blood pressure and diabetes, both established risk factors for cardiovascular mortality.

One potential explanation for these ethnic differences in disease incidence may be that BMI does not indicate features of body composition (such as body weight derived from lean or fat mass) or the distribution of body fat, which may differ across ethnicities with unique associations to risk [5]. To understand differences in the risk of cardiovascular disease (CVD), we need to understand how adiposity relates to intermediate cardiovascular risk factors across ethnic groups. However, large-scale studies investigating the association of body composition with risk factors for CVD across ethnic groups are lacking. Current evidence comes from small studies, often restricted to a single ethnic group, where the role of chance could skew the magnitude of associations.

This study compared measures of anthropometry and body composition with major established risk factors for cardiovascular disease measured at recruitment in two large prospective population-based cohort studies: TMC and UK Biobank. Despite the strength of UK Biobank’s 0.5 million participants, the cohort is limited by the lack of ethnic diversity, with 94% of participants from a White ethnic background. By contrast, reflecting the ethnic diversity of the Malaysian population, TMC is comprised of three major ethnic groups − Malay (44%), Chinese (33%) and Indian (15%) [2]. This allows for the largest comparison to date of anthropometry and body composition with risk factors for cardiovascular disease across multiple ethnic populations.

## Methods

### The Malaysian Cohort (TMC)

TMC recruited 106,527 healthy adults (i.e., without debilitating illness) aged 35–70 between 2006–2012 from rural and urban areas across Malaysia. Cluster sampling across 75 (of 103) rural settlements in Malaysia recruited 19,467 participants (75.1% response rate), whereas voluntary participation in urban areas recruited participants through advertisements and publicity campaigns [2]. Indians and Chinese were oversampled to allow reliable ethnic comparisons. Participants were interviewed at baseline about demographic and lifestyle characteristics, and medical history. Biophysical measurements were also taken, as were fasting blood samples. The Universiti Kebangsaan Malaysia Research Ethic Committee (UKM REC) (UKM 1.5.3.5/244/FF-2015-389) approved this study and informed consent was collected from all participants.

### UK Biobank

UK Biobank recruited 502,619 adults aged 40–69 between 2006–2010 who lived within 25 miles of 22 assessment centres located around major cities throughout England, Scotland, and Wales (response rate = 5.5%) [6]. Participants completed an electronic questionnaire about their sociodemographic, lifestyle and health-related characteristics, provided non-fasting blood samples, and had blood pressure and anthropometry recorded. Ethical approval was obtained from the North-West Multi-Centre Research Ethics Committee (REC reference: 11/NW/03820) and all participants provided written informed consent.

### Anthropometry and body composition

Fat mass and appendicular lean mass were measured using bioelectrical impedance analysis (BIA) in both cohorts. TMC used the multi-frequency InBody 720 system (Biospace, South Korea) and UK Biobank used the Tanita BC418MA single frequency segmental body-composition analyser (Tanita, Tokyo, Japan). In both cohorts, participants placed their bare feet on the analyser platform and gripped the metal handles; body fluid or hydration status was not measured nor controlled in either cohort [7]. “Appendicular lean mass” was indicated by the appendicular skeletal muscle mass (kg; the summed predicted muscle mass from the four limbs) as this region is the most modifiable by lifestyle factors such as exercise and potentially less confounded by concomitant fat mass in the trunk region [8]. Fat mass (kg) was derived from the body-composition analyser for the whole body. BMI was used as a measure of general adiposity and was measured in both cohorts as weight (kg) divided by the squared height (m). In TMC, height and weight were derived as the average of three measurements obtained from a Seca weight scale (SECA, Jerman) and Harpenden stadiometer (Holtain Limited, UK). In UK Biobank, height was measured once using a Seca 240 cm device (SEXA, Jerman) and weight was collected from the BIA device described above. Waist circumference (WC) was used as a measure of central adiposity, and was measured in both cohorts at the umbilicus over non-obstructive clothing using a tape measure. Additional analyses on waist-to-height ratio are included in Supplemental Table 2.

### Cardiovascular risk factors

Systolic blood pressure (SBP; mmHg) in TMC was measured three times using the OMRON HEM-907 model and measured twice in UK Biobank using an OMRON HEM-7015IT digital sphygmomanometer (Omron, Japan). The mean of all available measurements was used. In rare cases where the digital sphygmomanometer was unable to obtain a reading, a manual sphygmomanometer was used. Blood lipids were measured as detailed elsewhere [2, 9]; analyses were restricted to low-density lipoprotein (LDL-C; mmol/L) and triglycerides (mmol/L) due to their causal relevance for CVD risk [10].

Glycated haemoglobin (HbA1c; mmol/mol) was measured in UK Biobank in frozen packed red blood cells by Bio-Rad Variant II Turbo analyser using high-performance liquid chromatography (Bio-Rad Lab. Inc). In TMC, HbA1c (%) was measured using the high-performance liquid chromatography (HPLC) in the Variant™ II Turbo machine (Bio-Rad Laboratories Inc, USA). HbA1c measurements were introduced into the TMC study protocol later during recruitment, so measurements are only available on a subsample (*N* = 12,210; 11.5%; see Supplementary Table 1 for a comparison with the full cohort).

### Statistical analysis

The UK Biobank cohort was restricted to those of a White ethnicity and TMC to Malay, Chinese and Indian. To limit reverse causality, participants with self-reported prevalent diseases at baseline that could influence body composition were excluded: history of CVD, chronic bronchitis, hyperthyroidism, chronic hepatitis, and cancer within 5 years prior to the baseline survey. Participants were additionally excluded if they were outside the age range of 40–70 years, were pregnant, or had missing data on BIA measures. This left 30,271 Malay; 25,296 Chinese; 10,865 Indian; and 413,737 White participants. Analyses of SBP further excluded participants taking blood-pressure lowering medication, while analyses of lipid measures excluded participants taking lipid-lowering medications. Analyses of HbA1c excluded participants with a prior history of diabetes (Supplementary Fig. e1).

Linear regression was used to calculate age-adjusted means of fat mass, appendicular lean mass (adjusted for height) and WC by sex- and ethnicity-specific deciles of BMI, and of cardiovascular risk factors by sex- and ethnicity-specific quintiles of each body composition measure (Supplemental Figs. e2–e5).

Since the associations were approximately linear within each sex-by-ethnicity group, measures of body composition were included in the models as continuous variables to give the change in cardiovascular risk factor per unit change in body composition. BMI was presented as a 5 kg/m^2^ change to allow comparability with previous research [4]. Associations with body composition were compared to those with BMI by scaling the body composition measures to the same SD unit change. Scaling factors were based on the UK Biobank SDs since it had the largest sample size. For example, the BMI SD in UK Biobank males was 4.2 kg/m^2^, so a 5 kg/m^2^ change represents a 1.2 SD change. Therefore, we estimated a change in fat mass equivalent to a 1.2 SD change (approximately 10 kg). Analyses were also standardised to a sex- and- ethnic- specific 1 SD change to aid further comparisons of slopes across ethnic groups and are presented in the Supplement (Supplemental Figures e6-e9).

Linear regression models were adjusted for age (5-year age groups), height (cm), education (primary [no qualifications (UK) or through age 13 (TMC)], secondary [until age 16/17], tertiary [i.e., higher education]), smoking status (never/former vs current), alcohol intake (none, former, low intake or high intake in UK; and none or any in TMC due to low frequencies of drinking in Malaysia), and physical activity (Metabolic Equivalent Hours [MET] < 10 hr/wk, 10–50 hr/wk, 50+ hr/wk; as measured by the International Physical Activity Questionnaire) [11]. To assess the independent relevance of body composition measures, models of WC were additionally adjusted for BMI, and models of fat mass and appendicular lean mass were mutually adjusted. There were no violations of model assumptions.

Analyses were conducted using Stata version 15 (Stata Corp, TX, United States) and figures were constructed using R 3.5.2 (R Core Team, Vienna, Austria).

## Results

The mean age was 51.4 years in TMC (Malay 51.6, Chinese 51.3, and Indian 50.8) and 56.3 years in the UK Biobank, with 59% and 55% female, respectively. BMI (kg/m^2^) was lowest among the Chinese (24.7 [3.4]), similar in Indian (26.0 [3.8]) and Malay (26.1 [3.8]), and highest in White men (27.8 [4.2]) (Table 1). Among women, BMI (kg/m^2^) was also lowest among Chinese (23.8 [3.8]), but higher in Malay (27.4 [4.5]) and Indian (27.3 [4.4] than White (27.0 [5.1]). Similar to women, Chinese men had the lowest fat mass (18.8 [6.6] kg) and Indian men the highest (22.4 [7.5] kg). For appendicular lean mass, small differences were reported across ethnic groups in TMC, although Indian men and women had the lowest means. However, the average amount of lean mass was much larger in White adults than in TMC, with approximately 7 kg more in men and 5 kg more in women.

**Table 1:** Baseline characteristics (mean (SD)) of UK Biobank and The Malaysian Cohort.

	MEN			
	White (*n* = 184,433)	Malay (*n* = 13,011)	Chinese (*n* = 9614)	Indian (*n* = 4875)
Age, years	56.4 (8.1)	52.6 (6.8)	52.0 (7.0)	51.4 (6.7)
% Male	44.6	43.0	38.0	44.9
Anthropometry & body composition				
Weight, kg	86.2 (14.1)	70.9 (11.5)	69.8 (10.5)	73.3 (11.4)
Height, cm	176.0 (6.7)	164.7 (5.9)	168.0 (5.7)	167.7 (6.1)
Body mass index, kg/m^2^	27.8 (4.2)	26.1 (3.8)	24.7(3.4)	26.0 (3.8)
Total fat mass, kg	22.2 (8.2)	20.8 (7.5)	18.8 (6.6)	22.4 (7.5)
Appendicular lean mass, kg	28.8 (4.0)	21.0 (2.9)	21.5 (3.0)	21.0 (2.9)
Waist circumference, cm	96.8 (11.1)	88.1 (10.1)	86.7 (9.3)	92.0 (10.0)
Cardiovascular risk factors				
SBP, mmHg	141 (17)	130 (17)	130 (17)	129 (17)
LDL cholesterol, mmol/L	3.5 (0.8)	4.0 (1.0)	3.6 (1.0)	3.7 (0.9)
Triglycerides, mmol/L	2.0 (1.2)	1.8 (1.3)	1.7 (1.3)	1.7 (1.0)
HbA1c, % *	5.4 (0.6)	5.8 (1.0)	5.7 (0.7)	5.9 (0.9)
Lifestyle (*n*, %)				
Current drinkers	175,785 (95.3)	321 (2.5)	1,689 (17.6)	777 (15.9)
Current smokers	21,727 (11.8)	4,880 (37.5)	2,473 (25.7)	1,110 (22.8)
Moderate physical activity	85,042 (46.1)	3,088 (23.7)	2,431 (25.3)	1,520 (31.2)
Tertiary education	108,146 (58.6)	2,497 (19.2)	1,954 (20.3)	828 (17.0)

	WOMEN			
	White (*n* = 229,304)	Malay (*n* = 17,260)	Chinese (*n* = 15,682)	Indian (*n* = 5,990)
Age, years	56.3 (8.0)	50.9 (6.3)	50.9 (6.8)	(50.3) 6.5
% Female	55.4	57.0	62.0	55.1
Anthropometry & body composition				
Weight, kg	71.3 (13.8)	64.0 (11.2)	58.1 (9.8)	65.1 (11.0)
Height, cm	162.7 (6.2)	152.9 (5.4)	156.2 (5.4)	154.4 (5.7)
Body mass index, kg/m^2^	27.0 (5.1)	27.4 (4.5)	23.8 (3.8)	27.3 (4.4)
Total fat mass, kg	26.8 (9.9)	26.5 (8.0)	21.1 (6.7)	28.0 (7.9)
Appendicular lean mass, kg	19.5 (2.5)	15.0 (2.5)	14.8 (2.6)	14.6 (2.5)
Waist circumference, cm	84.3 (12.4)	85.0 (11.1)	78.7 (9.5)	86.9 (10.3)
Cardiovascular risk factors				
SBP, mmHg	135 (19)	129 (19)	127 (19)	126 (19)
LDL cholesterol, mmol/L	3.7 (0.9)	3.9 (1.1)	3.5 (0.9)	3.6 (0.9)
Triglycerides, mmol/L	1.5 (0.9)	1.4 (1.0)	1.2 (0.8)	1.4 (0.9)
HbA1c, % *	5.4 (0.5)	5.7 (0.9)	5.6 (0.6)	5.8 (0.9)
Lifestyle (*n*, %)				
Current drinkers	211,610 (92.3)	13 (0.1)	462 (2.9)	31 (0.5)
Current smokers	19,611 (8.6)	86 (0.5)	350 (2.2)	38 (0.6)
Moderate physical activity	109,023 (47.6)	4,193 (24.3)	4,710 (30.0)	2,069 (34.5)
Tertiary education	122,936 (53.6)	2,335 (13.5)	2,524 (16.1)	607 (10.1)

*HbA1c measurements available in *N* = 4639 men and *N* = 6859 women in the Malaysian Cohort. The scale of HbA1c in UK Biobank (mmol/mol) was converted to the same units as TMC (%) using the formula: (HbA1c(mmol/mol)/10.929) + 2.15.

**Fig. 1:**
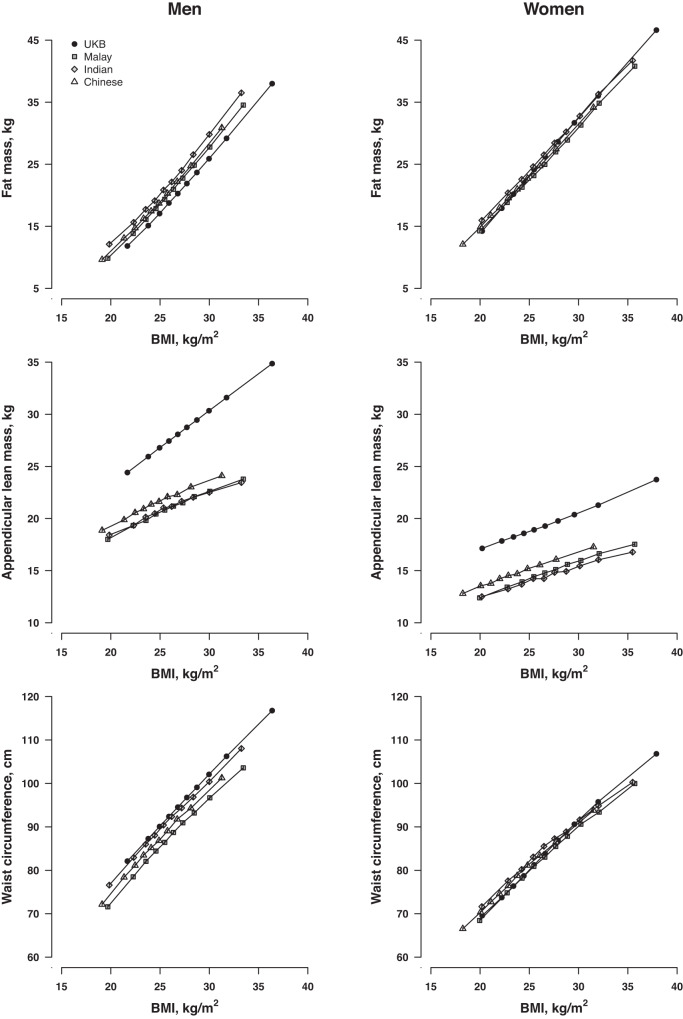
Adjusted means of fat mass, lean mass and waist circumference by body mass index (BMI) deciles across ethnicities, adjusted for age and height (lean mass only).

Fat mass for a given BMI was generally equivalent across ethnicities for women (Fig. 1), although Indian men had approximately 2–3 kg more fat mass than White men across the spectrum of BMI, with Malay and Chinese men intermediate. Once adjusted for height, Chinese participants had 1–2 kg more appendicular lean mass than Malay or Indian, although this was still about 5 kg less lean mass than White men and women. The disparity in appendicular lean mass increased further among White men with BMI ≥ 30 kg/m^2^. Women had a similar WC across all ethnic groups for each given BMI, but White and Indian men had approximately a 3–5 cm greater WC than Malay men at BMI > 27 kg/m^2^.

### Body mass index (BMI)

**Fig. 2:**
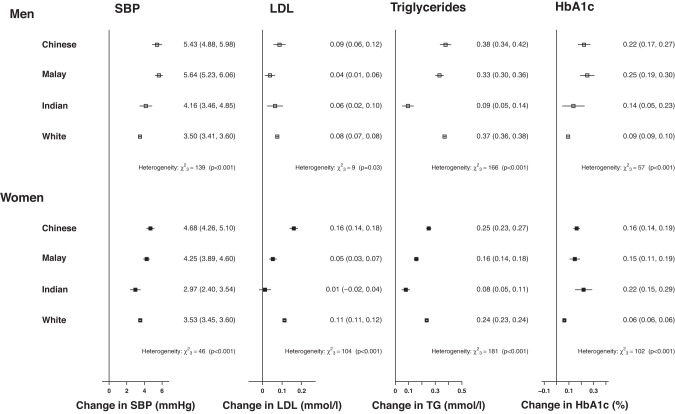
Fully adjusted associations of 5 kg/m^2^ higher body mass index (BMI) with cardiovascular disease risk factors. SBP systolic blood pressure, LDL low-density lipoprotein, TG triglycerides. Associations are fully adjusted for age, height, education, physical activity, smoking status, alcohol intake.

For each 5 kg/m^2^ higher BMI (Fig. 2), the greatest increase in SBP was reported for Chinese and Malay men (~5 mmHg) and weakest for the White men (3.5 mmHg, 95% CI: 3.4–3.6,). Small increases in LDL-C were similar across all male ethnic groups, but strongest in Chinese women (0.16 mmol/L, 0.14–0.18) and null in Indian women (0.01 mmol/L,−0.02–0.04). The association of BMI with triglycerides was notably weaker in both Indian men and women compared to the other groups (~0.09 vs ~0.25–0.38 mmol/L). Chinese and Malay men and Indian women reported similarly strong associations between BMI and HbA1c (~0.22% higher HbA1c), with weaker associations for White adults.

### Fat mass and appendicular lean mass

**Fig. 3:**
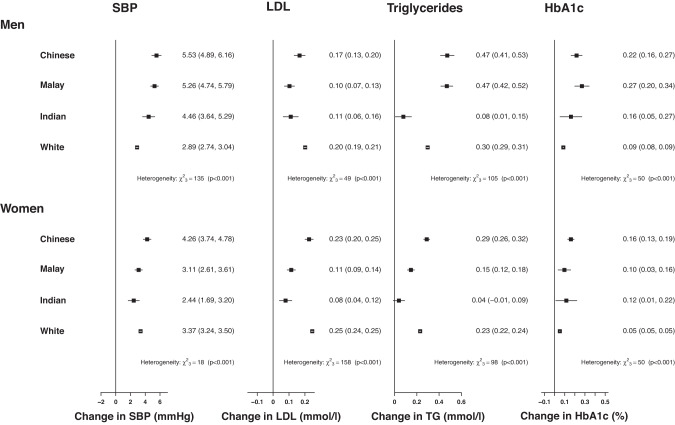
Fully adjusted associations of 10 kg higher fat mass with cardiovascular disease risk factors. SBP systolic blood pressure, LDL low-density lipoprotein, TG triglycerides. Associations are fully adjusted for age, height, education, physical activity, smoking status, alcohol intake and lean mass.

There were similar patterns across ethnic groups for a 10 kg higher fat mass (adjusted for appendicular lean mass) as there was for BMI (Fig. 3). However, the absolute mean changes were marginally weaker between fat mass and SBP than for BMI (e.g., 2.9 vs 3.5 mmHg for White men). Conversely, the average increase in mean LDL-C was nearly twice as strong for fat mass as for BMI for most ethnic groups (e.g., 0.17 vs 0.09 mmol/L for Chinese men).

**Fig. 4:**
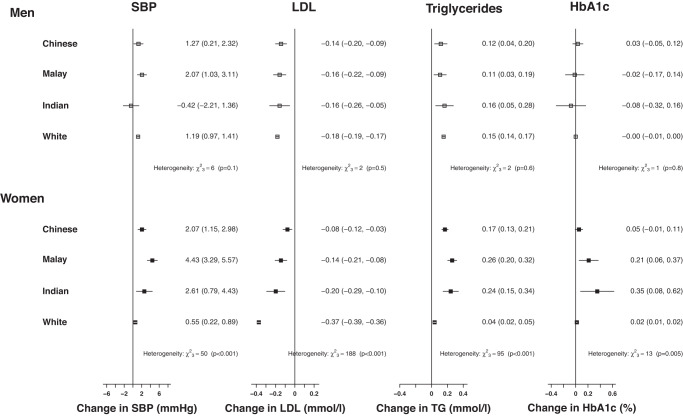
Fully adjusted associations of 6 kg higher lean mass with cardiovascular disease risk factors. SBP systolic blood pressure, LDL low-density lipoprotein, TG triglycerides. Associations are fully adjusted for age, height, education, physical activity, smoking status, alcohol intake and fat mass.

Associations between appendicular lean mass (adjusted for fat mass) and cardiovascular risk factors (Fig. 4) were distinct from BMI. Appendicular lean mass was weakly positively associated with SBP (1–2 mmHg higher per 6 kg higher lean mass) in most male ethnic groups (except a null association for Indian men), although this association was nearly twice as strong in all female Malaysian ethnicities, particularly Malay women. Higher appendicular lean mass was positively associated with triglycerides and inversely associated with LDL-C to a similar extent across all sex and ethnic groups, except for White women. Associations between appendicular lean mass and HbA1c were null for most sex and ethnic groups except for Malay (0.21; 95% CI: 0.06–0.37) and Indian women (0.35; 95% CI: 0.08–0.62), where the associations were stronger than they were for BMI.

### Waist circumference

**Fig. 5:**
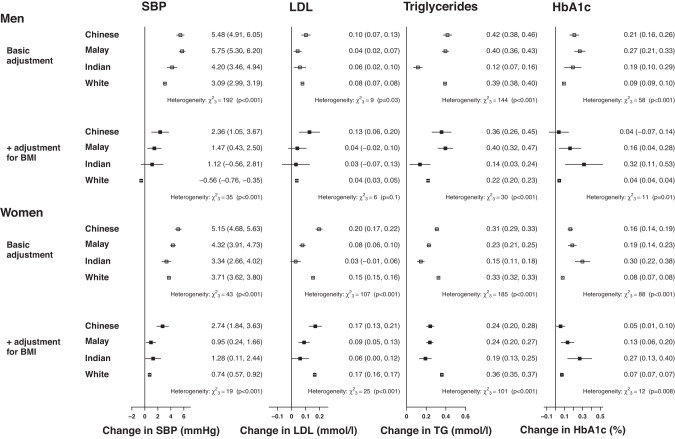
Fully adjusted associations of 14 cm higher waist circumference with cardiovascular disease risk factors. SBP systolic blood pressure, LDL low-density lipoprotein, TG triglycerides. Associations are fully adjusted for age, height, education, physical activity, smoking status, alcohol intake. Models are presented without and with mutual adjustment for body mass index.

Before adjusting for BMI, the associations between a 14 cm greater waist circumference and all cardiovascular risk factors were generally equivalent to those with BMI for men but were slightly stronger for women (Figs. 2 and 5). After mutually adjusting for BMI, however, the associations between WC and SBP were largely or wholly attenuated for all ethnic groups (Fig. 5). Associations between WC, LDL-C and triglycerides were not substantively affected by adjustment for BMI. However, adjustment for BMI had diverse effects on the associations between WC with HbA1c across sex and ethnic groups. Associations were wholly attenuated for Chinese participants, partly attenuated for Malay participants, and strengthened for Indian men but unaffected for Indian women. Overall, fully adjusted associations between WC, SBP and lipids tended to be strongest in the Chinese groups and weakest in the Indian groups, whereas this pattern was reversed for HbA1c.

## Discussion

In the largest ethnic comparison of adiposity, body composition and cardiovascular risk factors study to date, we observed distinctly different patterns with CVD risk factors across ethnic groups despite generally small differences in body composition at a given BMI. BMI and fat mass had similar positive associations with SBP and HbA1c (although stronger overall in Malaysian ethnicities than White); but the associations with lipids were generally stronger for fat mass. A notable exception was for Indian men and women for whom there was little association of either BMI or fat mass with triglycerides. Contrasting associations across CVD risk factors were observed for appendicular lean mass, with no evidence in men of differences across ethnic groups. However, among women, associations with appendicular lean mass were particularly strong in Malay and Indian women, with positive associations that were greater than those for fat mass or BMI. Adjustment for BMI did not impact associations between WC and lipids, but it largely attenuated associations with SBP and produced diverse effects on associations with HbA1c across the sex- and ethnic-groups.

Previous research has documented different obesity-related risks across ethnic groups, with South Asians generally at a higher risk for diabetes but a lower risk for CVD than Caucasians at similar levels of BMI [2, 3, 12]. BMI has been criticised as a measure of adiposity since it does not indicate potentially important characteristics of body composition for disease risk, such as the proportion of fat and lean mass, or fat distribution [13, 14]. However, this study observed distinctly different patterns of body composition and CVD risk factors across ethnic groups despite generally small differences in body composition at a given BMI. Chinese men and women were found to have the lowest average BMI, fat mass and SBP (by nearly 10 mmHg compared to Whites), but their associations of adiposity with CVD risk factors (particularly SBP) tended to be the strongest of any group (e.g., 5 mmHg SBP per 5 kg/m^2^ BMI or 10 Kg fat mass). Other research has also reported that Chinese men had stronger relationships with SBP, fasting glucose and blood lipids than White men for a given BMI, suggesting they were more prone to the metabolic effects of obesity [15]. Interestingly, the strong relationship between BMI and SBP for the Chinese in this study was still weaker than associations reported from large-scale studies of Chinese adults from mainland China (8.3 mmHg per 5 kg/m^2^ BMI), suggesting there may be environmental interactions for cardiovascular risk that future research should investigate [16, 17].

Even though BMI as a measure of adiposity has been criticised for failing to distinguish between types of tissue mass, ethnic comparisons showed broadly similar patterns for fat mass and BMI (although lipids were slightly more strongly associated with fat mass). Conversely, associations with appendicular lean mass were distinct from those reported with BMI and not consistently beneficial. The positive association between lean mass and SBP has been documented before across White and Non-White ethnicities, but this study reported a novel finding that in Malay and Indian women the deleterious associations of SBP, triglycerides and HbA1c with appendicular lean mass were generally stronger than those with BMI or fat mass [18, 19]. Previous research on a Malay population in Malaysia found higher metabolic risks at lower levels of BMI and WC than recommended by international diagnostic criterion, suggesting other elements of body composition were important for metabolic risk [20]. Current evidence is equivocal regarding the role of lean mass in cardiometabolic health, with large prospective studies reporting both increased and decreased risks of incident CVD with greater lean mass [14, 21, 22]. Theories suggest that muscle tissue is the main depot for glucose uptake and clearance, entailing that greater lean mass should improve insulin sensitivity. However, meta-analyses of resistance training interventions in participants with diabetes indicated that improvements in glycaemic control were seen alongside improvements in strength, without gains in absolute lean mass [21, 23]. This suggests future studies need to look more closely at muscle quality in relation to cardiovascular health, such as fibre typology and fat accumulation, particularly as previous research has reported that south Asians may have higher intermuscular fat than BMI-matched White or Chinese groups [21, 24]. Differences in muscle quality may further differ by sex-specific ethnic groups, given the particularly strong associations of lean mass with triglycerides and HbA1c for Malay and Indian women in this study. This could be an important source of heterogeneity for metabolic health that needs to be examined.

Another novel finding from this study was that associations of WC with HbA1c were largely attenuated by adjustment for BMI in Chinese adults, but were less affected in the Malay and were strengthened in Indian men. Few studies have compared associations of general and central adiposity across ethnicities. One study on 2500 adults from different ethnicities in the London SABRE study found that central adiposity, particularly visceral adipose tissue, was a stronger risk factor for diabetes in south Asian than European men [25]. Likewise, Indian men and women in this study had the strongest associations between WC and HbA1c of any ethnic group. Such differences may be due to adipocyte morphology, with suggestions that south Asians may have a lower capacity to store fat in subcutaneous fat depots, so excess fat more readily overflows into ectopic compartments that increase metabolic impairment [26]. However, this theory contradicts the markedly weaker relationships between adiposity and triglycerides for Indian men and women in this study, as an increase in liver fat accumulation is often accompanied by elevated triglycerides [27]. Such weak associations are also interesting as elevated triglycerides are generally associated with insulin resistance and diabetes, with Indian adults reporting elevated risks of both compared to other ethnicities [28, 29]. In the future, incorporation of genetic data would help elucidate the independent relevance of different anthropometric and body composition measures across ethnic groups. For example, a previous sub-study in TMC suggested there was a gradient in genetic risk scores for type II diabetes across ordered strata of BMI, with the genetic risk score having progressively larger effects across decreasing levels of BMI. However, that study was too small to detect differences across ethnic groups and genetic evidence in multi-ethnic populations for other measures of body composition like ectopic fat is currently lacking [30, 31].

A clear strength of this research is that it is the largest study to date with global multi-ethnic comparisons of detailed measures of body composition and cardiovascular risk factors, so chance findings due to small sample sizes between ethnic- and sex- specific groups is less likely. Furthermore, the Malaysian and UK studies began recruitment around the same time, and had harmonised measurements on many covariates. TMC collected fasting blood samples from their participants, whereas UK Biobank did not, which limits the comparisons of lipids between the two studies, although it still allows for comparisons within TMC. Additionally, while both studies assessed body composition using BIA, this was done using two different models, each with their own algorithms for estimating fat and lean mass. Such algorithms are patented and unavailable for comparison, but one previous study comparing the validity of two different bioimpedance machines (a single frequency Tanita model and a multi-frequency InBody model, akin to this study) on Taiwanese children suggested that both models had very high agreement with measurements from dual-energy x-ray absorptiometry (DXA) scans: the intraclass correlation coefficients were >0.94 for all estimates (except lean mass in boys) [32]. However, since the two different BIA models were not able to be calibrated to a gold standard measure in this study, any inferences on body composition should be limited to within-cohort comparisons. Furthermore, neither study was able to adjust the associations with BIA for hydration status, a key factor that can impact the measurements [32]. Future research should investigate if more detailed measurements of body composition across ethnicities, such as those from DXA, would produce similar associations.

The data used in this study was cross-sectional, so temporality and causality cannot be inferred. Even though a comprehensive list of prevalent diseases were excluded in both datasets to limit reverse causality, there is still the possibility that prevalent subclinical disease could be influencing both CVD risk factors and body composition when measurements were taken. Residual confounding is also possible due to both unmeasured confounders and to errors within measured confounders (e.g., self-reported smoking status and physical activity), meaning we have not fully adjusted for the true values of these confounders. In particular, dietary intake was not adjusted for, but since sequential adjustment for other lifestyle factors had little impact on the associations (data not shown), it is unlikely that adjustment for dietary intake would have made a substantive impact. Different relationships with body composition could also be due to environmental differences between and within countries for ethnic groups.

Overall, this study observed distinctly different patterns of adiposity and body composition with CVD risk factors across ethnic groups despite generally small differences in body composition at a given BMI. Chinese men and women had a smaller BMI and less fat mass, but the strongest associations with many risk factors. Meanwhile, Indian participants reported the strongest relationships between WC and HbA1c, particularly after adjustment for BMI, but notably weak associations between adiposity and triglycerides. There were consistently weak associations with appendicular lean mass across male ethnic groups, but positive relationships between lean mass and several risk factors were stronger in Malay and Indian women than for BMI. Despite these distinct patterns across ethnic groups, it is still not clear why marked differences in the risks for diabetes or CVD for a given BMI have been observed in different ethnic groups. The limitations of BIA and the as-yet unclear mechanisms linking aspects of body composition to cardiovascular disease suggest that more detailed measurements of regional fat and lean mass across ethnicities needs to be undertaken. Only once the mechanisms linking adiposity and body composition with disease aetiology are better understood can we start to engage with more targeted prevention strategies to help attenuate the increasing global burden of obesity-associated diseases.

## Research in context

### Evidence before this study

The global burden of obesity-related disease has been increasing over the last three decades, but the metabolic risks associated with adiposity differ between populations and are not completely understood. PubMed was searched for all papers up to July 2021 containing words related to (1) adiposity or body composition (e.g., body mass index, waist circumference, fat mass, muscle mass) (2), blood pressure, lipids, HbA1c, or glucose; and (3) South Asia, China, Malaysia or Europe. Studies were excluded if they studied children, adolescents, or elderly populations; and if they focused on weight maintenance, weight management or weight reduction. Nearly all studies focussed on a single ethnicity, and the few studies with multi-ethnic populations were small (*N* < 10,000) and mainly descriptive. No large-scale studies compared relative associations between ethnicities regarding anthropometry and body composition and cardiovascular disease (CVD) risk factors.

### Added value of this study

In the largest comparison to date of global multi-ethnic populations; with harmonised data on over 30,000 Malay, 25,000 Chinese, 10,000 Indian and 410,000 White Europeans; unique insights into metabolic health were observed. Chinese participants had lower absolute levels of adiposity but generally stronger deleterious relationships to CVD risk factors than Malay, Indian or White participants. Those of Indian descent had markedly weaker relationships between adiposity and triglycerides, but the strongest relationship between waist circumference and HbA1c. Associations with appendicular lean mass were not consistently beneficial, particularly for Malay and Indian women, among whom there were positive relationships with systolic blood pressure, triglycerides and HbA1c that were stronger than those for BMI.

### Implications of all the available evidence

There were distinct patterns in adiposity and body composition and CVD risk factors across sex and ethnic groups that do not explain observed variation in CVD rates across populations. The unclear mechanisms linking body composition to cardiovascular disease risk suggest that more detailed measurements of regional fat and lean mass across ethnicities needs to be undertaken. Only once the mechanisms underlying associations of adiposity and body composition with CVD are better understood can we start to engage with appropriately targeted prevention strategies to attenuate the increasing global burden of disease from obesity.

## Supplementary information

Supplemental Material

## Data Availability

All results from this analysis are returned to UK Biobank within 6 months of publication, at which point they can be made available to other researchers upon reasonable request. UK Biobank is an open access resource, and researchers can apply to use the dataset at http://ukbiobank.ac.uk/register-apply/. Data analysis in TMC is done by staff at Universiti Kebangsaan Malaysia but relevant tables and analytic code can be shared with researchers upon reasonable request. The study protocols are published online at https://www.ukbiobank.ac.uk/learn-more-about-uk-biobank/about-us and 10.1093/ije/dyu089. The statistical analysis plan and analytic code are available upon request to the corresponding author.
