# Supplementary material for: Body composition and risk factors for cardiovascular disease in global multi-ethnic populations
Source: Int J Obes (Lond). 2023 Jul 17;47(9):855–64. doi: 10.1038/s41366-023-01339-9 (PMC10439008; doi:10.1038/s41366-023-01339-9)

# SUPPLEMENTAL MATERIAL

## Body composition and risk factors for cardiovascular disease in global multi-ethnic populations

**Contents**

Body composition and risk factors for cardiovascular disease in global multi-ethnic populations ....... 1

Supplemental Table 1. Characteristics of the subsample of The Malaysia Cohort with HbA1c measurements available (N=12,031; 18.1%) ....... 2

Supplemental Table 2. Fully adjusted associations with risk factors for cardiovascular disease for a 1 SD increase in waist (cm)-to-height (cm) ratio....... 3

Supplemental Figure e1. Flow diagram of exclusions made to analytic samples of The Malaysia Cohort and UK Biobank....... 4

Supplemental Figure e2. Associations between body mass index (BMI) quintiles (adjusted for age) and cardiovascular risk factors across ethnicities....... 5

Supplemental Figure e3. Associations between fat mass quintiles (adjusted for age) and cardiovascular risk factors across ethnicities....... 6

Supplemental Figure e4. Associations between lean mass quintiles (adjusted for age) and cardiovascular risk factors across ethnicities....... 7

Supplemental Figure e5. Associations between waist circumference quintiles (adjusted for age) and cardiovascular risk factors across ethnicities....... 8

Supplemental Figure e6. Fully adjusted associations between cardiovascular risk factors a 1 sex- and ethnic- specific SD change in body mass index (BMI). ....... 9

Supplemental Figure e7. Fully adjusted associations between cardiovascular risk factors a 1 sex- and ethnic- specific SD change in fat mass. ....... 10

Supplemental Figure e8. Fully adjusted associations between cardiovascular risk factors a 1 sex- and ethnic- specific SD change in lean mass....... 11

Supplemental Figure e9. Fully adjusted associations between cardiovascular risk factors a 1 sex- and ethnic- specific SD change in waist circumference. ....... 12

**Supplemental Table 1. Characteristics of the subsample of The Malaysia Cohort with HbA1c measurements available (N=12,031; 18.1%), and the UK Biobank sample with measurements available (N=413,737).**

| | **MEN** | | | |
|---|---|---|---|---|
| | **White (n=184,433)** | **Malay (N=2,323)** | **Chinese (N=1,908)** | **Indian (N=716)** |
| Age, years | 56.4 (8.1) | 50.4 (6.9) | 52.5 (7.1) | 51.3 (6.8) |
| *Body composition* | | | | |
| Weight, kg | 86.2 (14.1) | 72.4 (11.3) | 69.8 (10.6) | 74.9 (12.0) |
| Height, cm | 176.0 (6.7) | 166.1 (5.8) | 168.5 (5.7) | 168.8 (6.3) |
| Body mass index, kg/m$^2$ | 27.8 (4.2) | 26.2 (3.8) | 24.6 (3.4) | 26.3 (4.1) |
| Total fat mass, kg | 22.2 (8.2) | 21.1 (7.4) | 18.6 (6.7) | 23.1 (8.1) |
| Lean mass, kg | 28.8 (4.0) | 28.6 (3.5) | 28.5 (3.5) | 28.9 (3.6) |
| Waist circumference, cm | 96.8 (11.1) | 88.3 (10.1) | 86.4 (9.4) | 92.7 (10.8) |
| *Cardiovascular risk factors* | | | | |
| SBP, mmHg | 141 (17) | 132.9 (18.5) | 133.7 (17.9) | 132.2 (17.7) |
| LDL cholesterol, mmol/L | 3.5 (0.8) | 4.0 (1.0) | 3.5 (0.9) | 3.7 (1.0) |
| Triglycerides, mmol/L | 2.0 (1.2) | 1.8 (1.2) | 1.7 (1.4) | 1.7 (1.0) |
| HbA1c, % * | 5.4 (0.6) | 5.9 (1.2) | 5.8 (1.0) | 6.2 (1.3) |
| *Lifestyle (n, %)* | | | | |
| Current drinkers | 175,785 (95.3) | 30 (1.3) | 321 (16.8) | 93 (13.0) |
| Current smokers | 21,727 (11.8) | 813 (35.0) | 481 (25.2) | 168 (23.5) |
| Moderate physical activity | 85,042 (46.1) | 980 (42.2) | 848 (44.4) | 303 (42.3) |
| Tertiary education | 108,146 (58.6) | 731 (31.5) | 545 (28.6) | 205 (28.6) |
| | **WOMEN** | | | |
| | **White (n=229,304)** | **Malay (n=2,839)** | **Chinese (n=3,335)** | **Indian (n=910)** |
| Age, years | 56.3 (8.0) | 49.5 (6.3) | 51.6 (6.8) | 50.0 (6.5) |
| *Body composition* | | | | |
| Weight, kg | 71.3 (13.8) | 64.8 (11.3) | 57.8 (9.8) | 66.0 (11.5) |
| Height, cm | 162.7 (6.2) | 154.3 (5.5) | 156.7 (5.5) | 155.5 (5.7) |
| Body mass index, kg/m$^2$ | 27.0 (5.1) | 27.2 (4.4) | 23.5 (3.8) | 27.3 (4.5) |
| Total fat mass, kg | 26.8 (9.9) | 26.9 (7.9) | 20.7 (6.7) | 28.3 (8.2) |
| Lean mass, kg | 19.5 (2.5) | 20.31 (2.9) | 19.8 (2.7) | 20.2 (2.9) |
| Waist circumference, cm | 84.3 (12.4) | 83.9 (11.0) | 78.4 (9.7) | 86.9 (10.7) |
| *Cardiovascular risk factors* | | | | |
| SBP, mmHg | 135 (19) | 130.8 (20.6) | 129.5 (20.3) | 129.3 (19.7) |
| LDL cholesterol, mmol/L | 3.7 (0.9) | 3.8 (1.0) | 3.5 (0.9) | 3.6 (0.9) |
| Triglycerides, mmol/L | 1.5 (0.9) | 1.3 (0.7) | 1.2 (0.7) | 1.3 (0.8) |
| HbA1c, % * | 5.4 (0.5) | 5.8 (1.1) | 5.6 (0.7) | 6.0 (1.3) |
| *Lifestyle (n, %)* | | | | |
| Current drinkers | 211,610 (92.3) | 6 (0.2) | 113 (3.4) | 7 (0.8) |
| Current smokers | 19,611 (8.6) | 23 (0.8) | 87 (2.6) | 4 (0.4) |
| Moderate physical activity | 109,023 (47.6) | 1,390 (49.0) | 1,793 (53.8) | 430 (47.3) |
| Tertiary education | 122,936 (53.6) | 818 (28.8) | 762 (22.9) | 158 (17.4) |

**Supplemental Table 2. Fully adjusted associations with risk factors for cardiovascular disease for a 1 SD increase in waist (cm)-to-height (cm) ratio.** [a]

| MEN | | Standard Deviation | SBP, mmHg | LDL, mmol/L | TRIGLYCERIDES, mmol/L | HbA1c, % |
|---|---|---|---|---|---|---|
| | Chinese | 0.06 | 3.63 (3.25, 4.01) | 0.07 (0.05, 0.09) | 0.28 (0.25, 0.30) | 0.05 (0.04, 0.06) |
| | Malay | 0.06 | 4.07 (3.74, 4.39) | 0.04 (0.02, 0.06) | 0.28 (0.26, 0.30) | 0.06 (0.05, 0.08) |
| | Indian | 0.06 | 2.95 (2.43, 3.48) | 0.05 (0.02, 0.08) | 0.08 (0.05, 0.12) | 0.04 (0.02, 0.07) |
| | White | 0.06 | 2.74 (2.66, 2.82) | 0.00 (-0.01, 0.00) | 0.32 (0.32, 0.33) | 0.14 (0.14, 0.15) |
| | | | | | | |
| WOMEN | | | | | | |
| | Chinese | 0.06 | 3.59 (3.27, 3.91) | 0.14 (0.12, 0.16) | 0.21 (0.19, 0.22) | 0.03 (0.02, 0.03) |
| | Malay | 0.07 | 3.41 (3.08, 3.74) | 0.07 (0.05, 0.08) | 0.18 (0.16, 0.20) | 0.04 (0.03, 0.05) |
| | Indian | 0.07 | 2.34 (1.84, 2.85) | 0.03 (0.00, 0.05) | 0.10 (0.08, 0.13) | 0.07 (0.05, 0.09) |
| | White | 0.08 | 3.51 (3.43, 3.58) | 0.08 (0.08, 0.08) | 0.31 (0.30, 0.31) | 0.12 (0.12, 0.12) |

[a] Adjusted for age, education, smoking, alcohol and physical activity. SBP= Systolic blood pressure; LDL=Low density lipoprotein; HbA1c=glycated haemoglobin.

**Supplemental Figure e1. Flow diagram of exclusions made to analytic samples of The Malaysia Cohort and UK Biobank.**

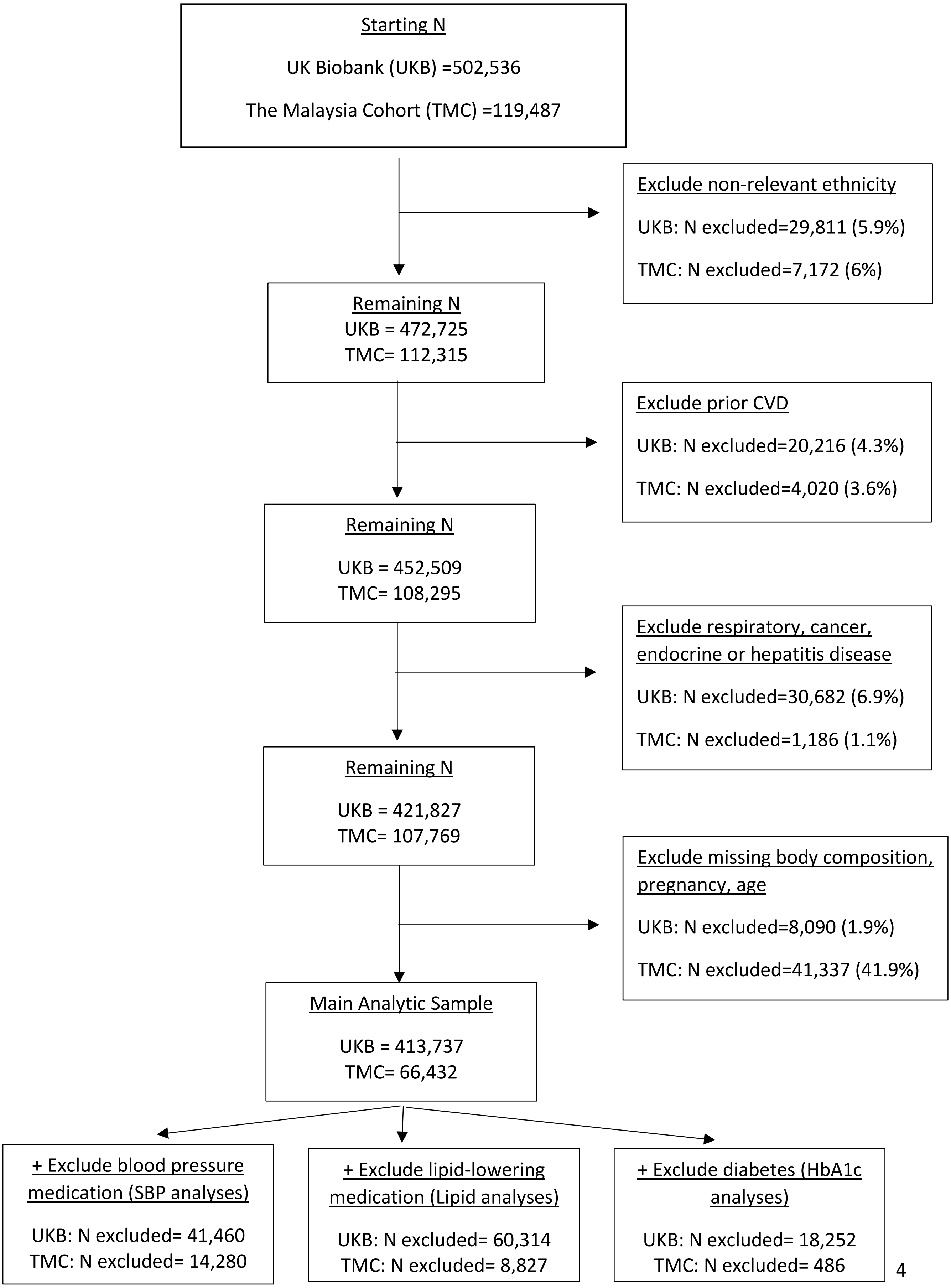

**Supplemental Figure e2. Associations between body mass index (BMI) quintiles (adjusted for age) and cardiovascular risk factors across ethnicities.**

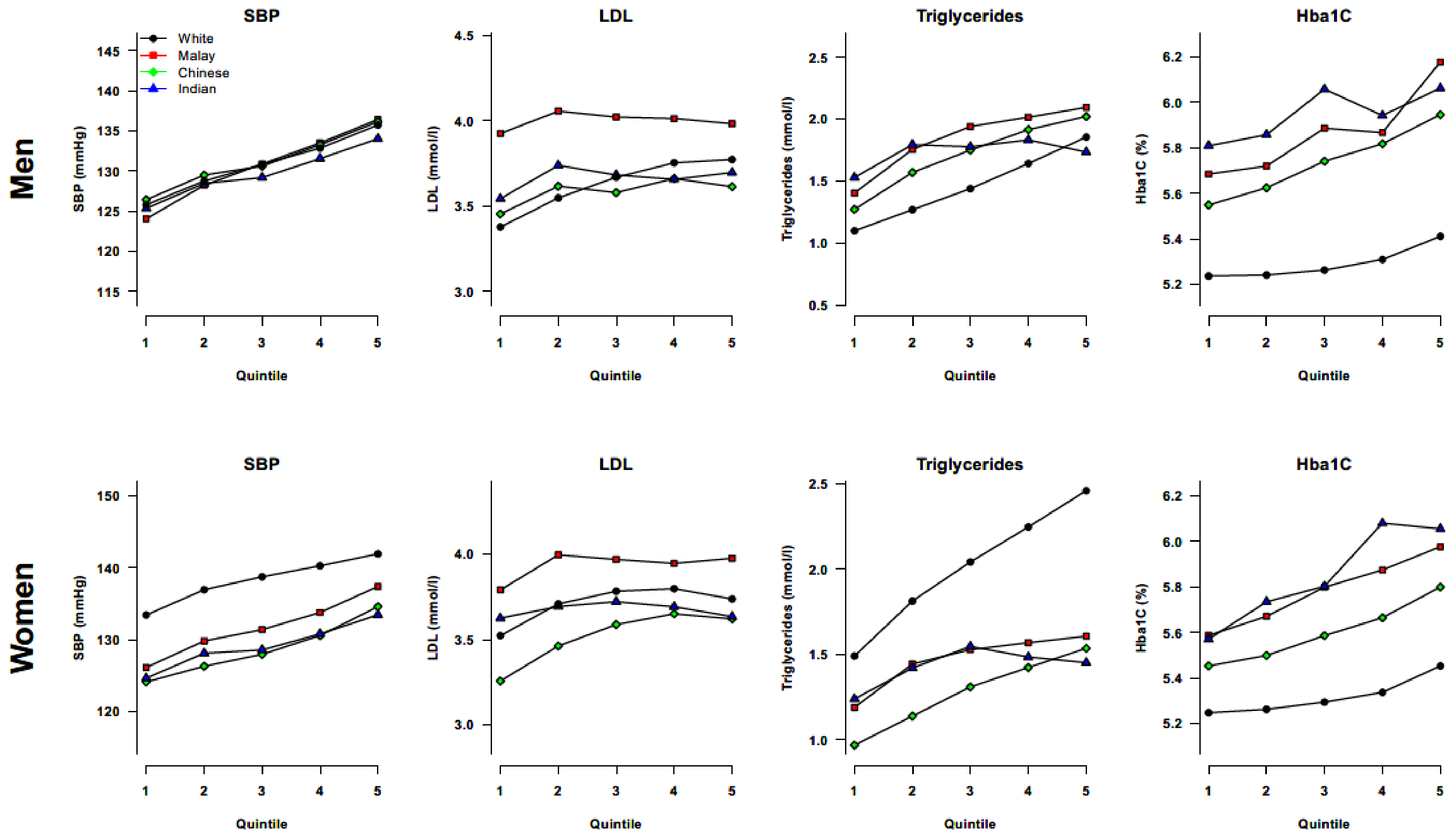

**Supplemental Figure e3. Associations between fat mass quintiles (adjusted for age) and cardiovascular risk factors across ethnicities.**

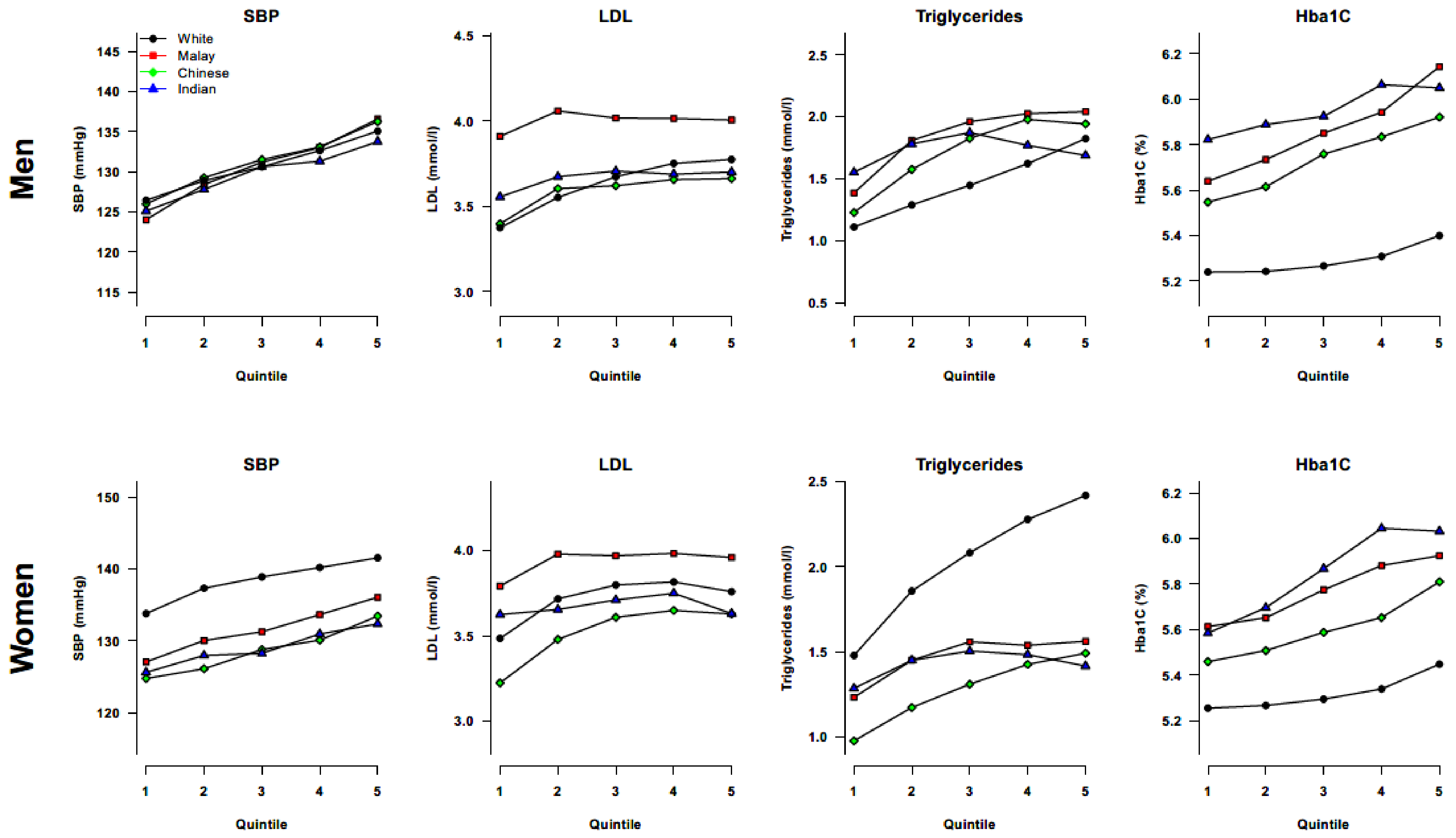

**Supplemental Figure e4. Associations between <u>lean mass</u> quintiles (adjusted for age) and cardiovascular risk factors across ethnicities.**

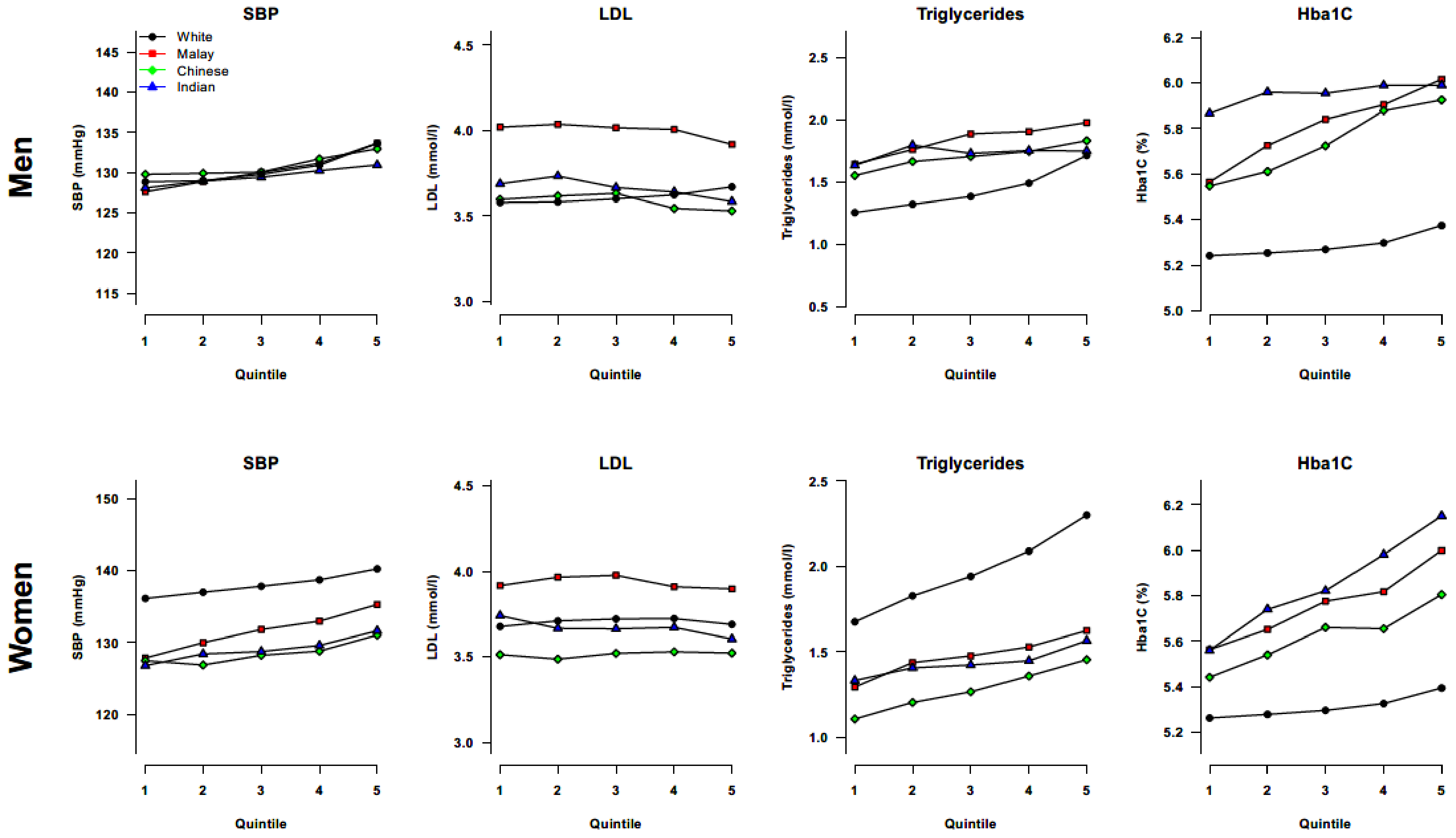

**Supplemental Figure e5. Associations between waist circumference quintiles (adjusted for age) and cardiovascular risk factors across ethnicities.**

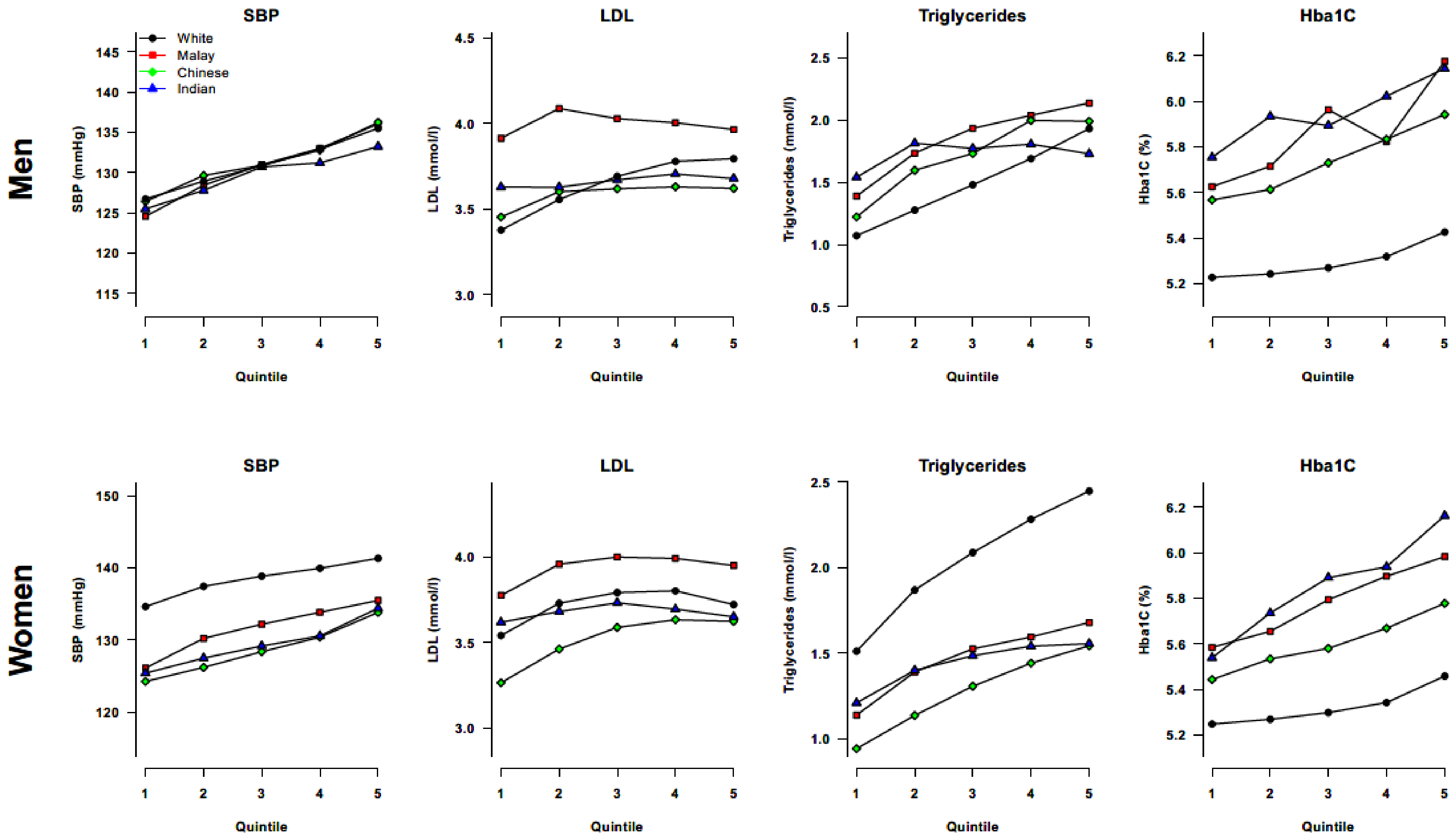

**Supplemental Figure e6. Fully adjusted associations between cardiovascular risk factors a 1 sex- and ethnic- specific SD change in <u>body mass index (BMI)</u>.**

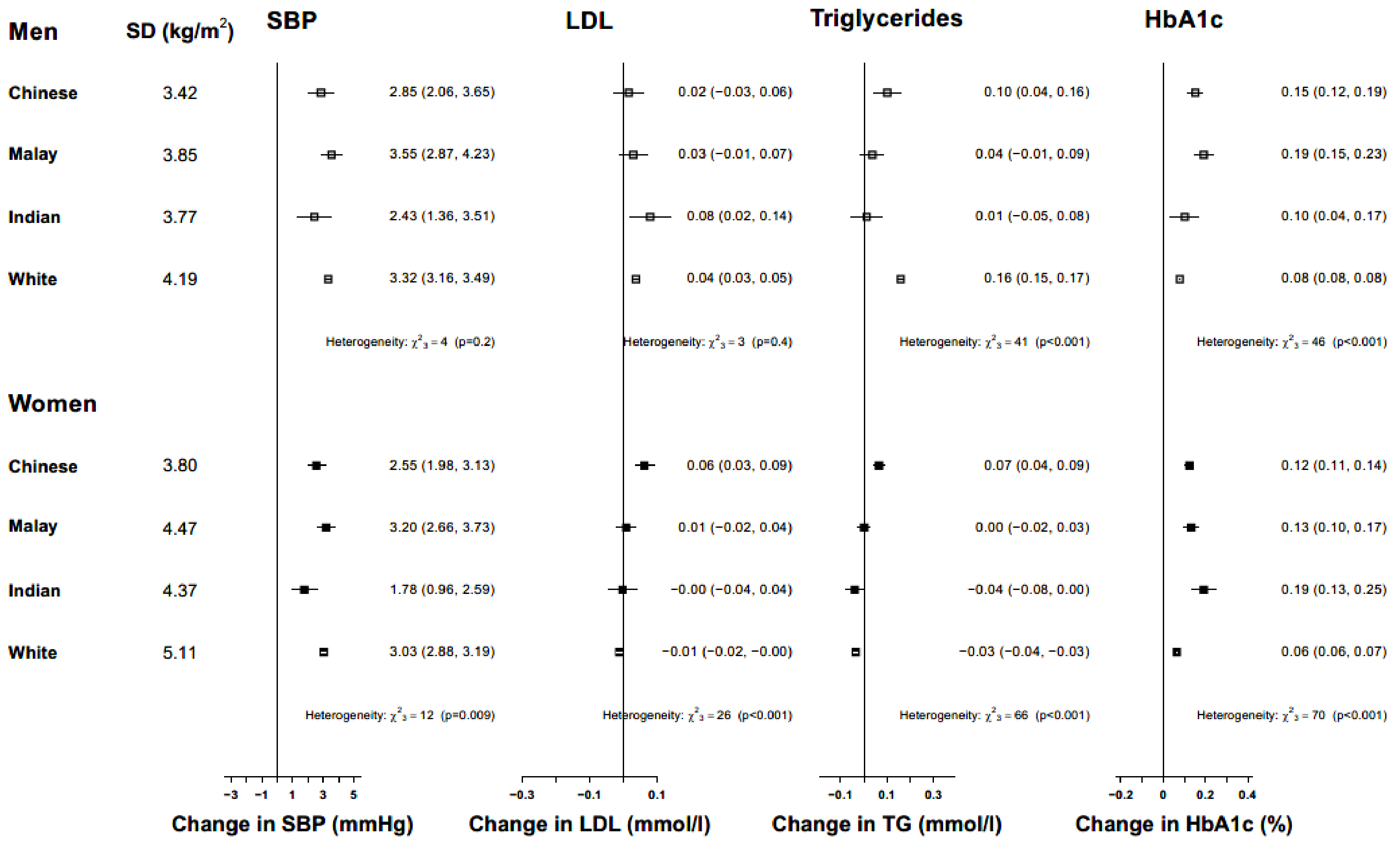

**Supplemental Figure e7. Fully adjusted associations between cardiovascular risk factors a 1 sex- and ethnic- specific SD change in <u>fat mass</u>.**

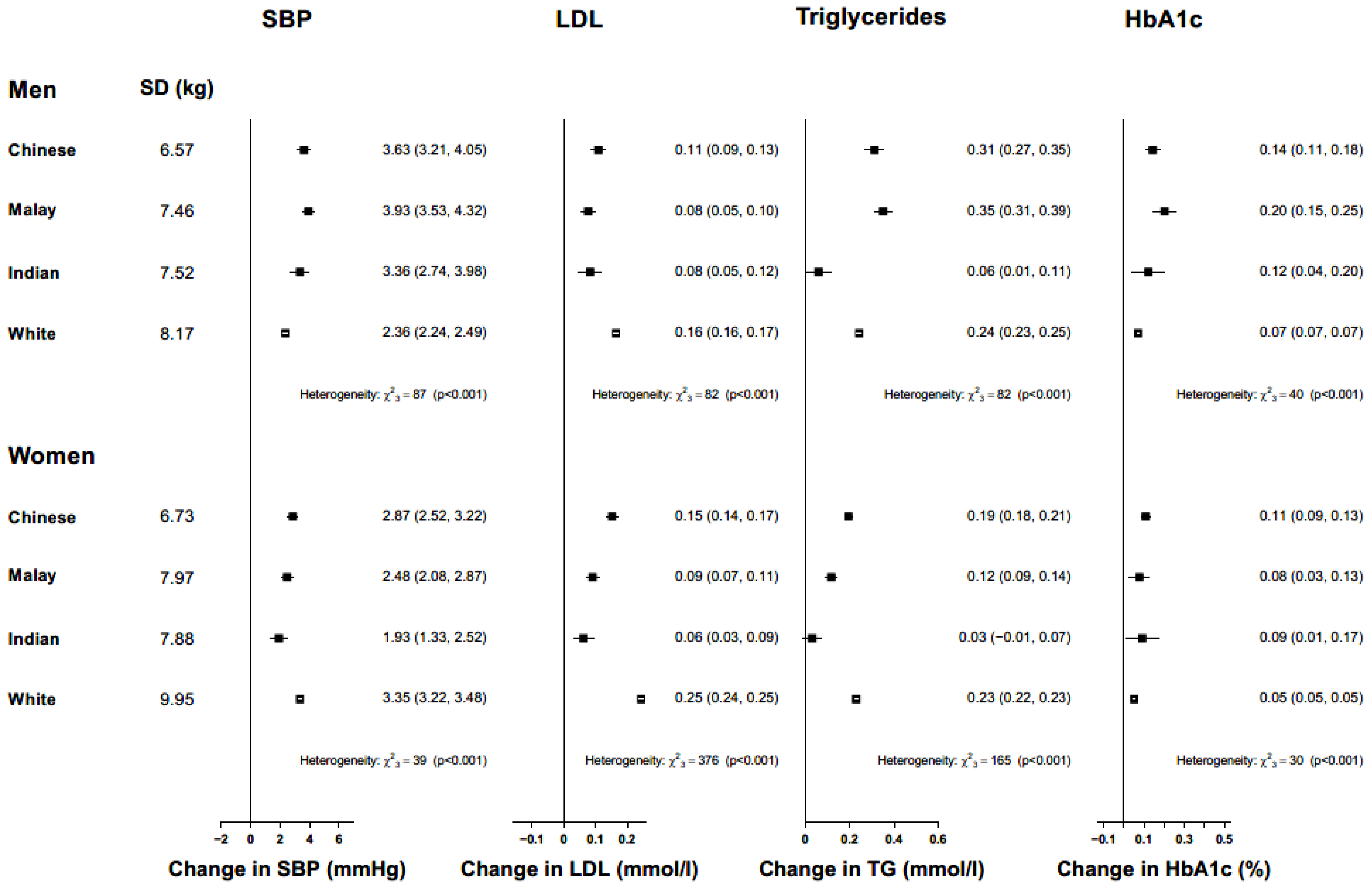

**Supplemental Figure e8. Fully adjusted associations between cardiovascular risk factors a 1 sex- and ethnic- specific SD change in lean mass.**

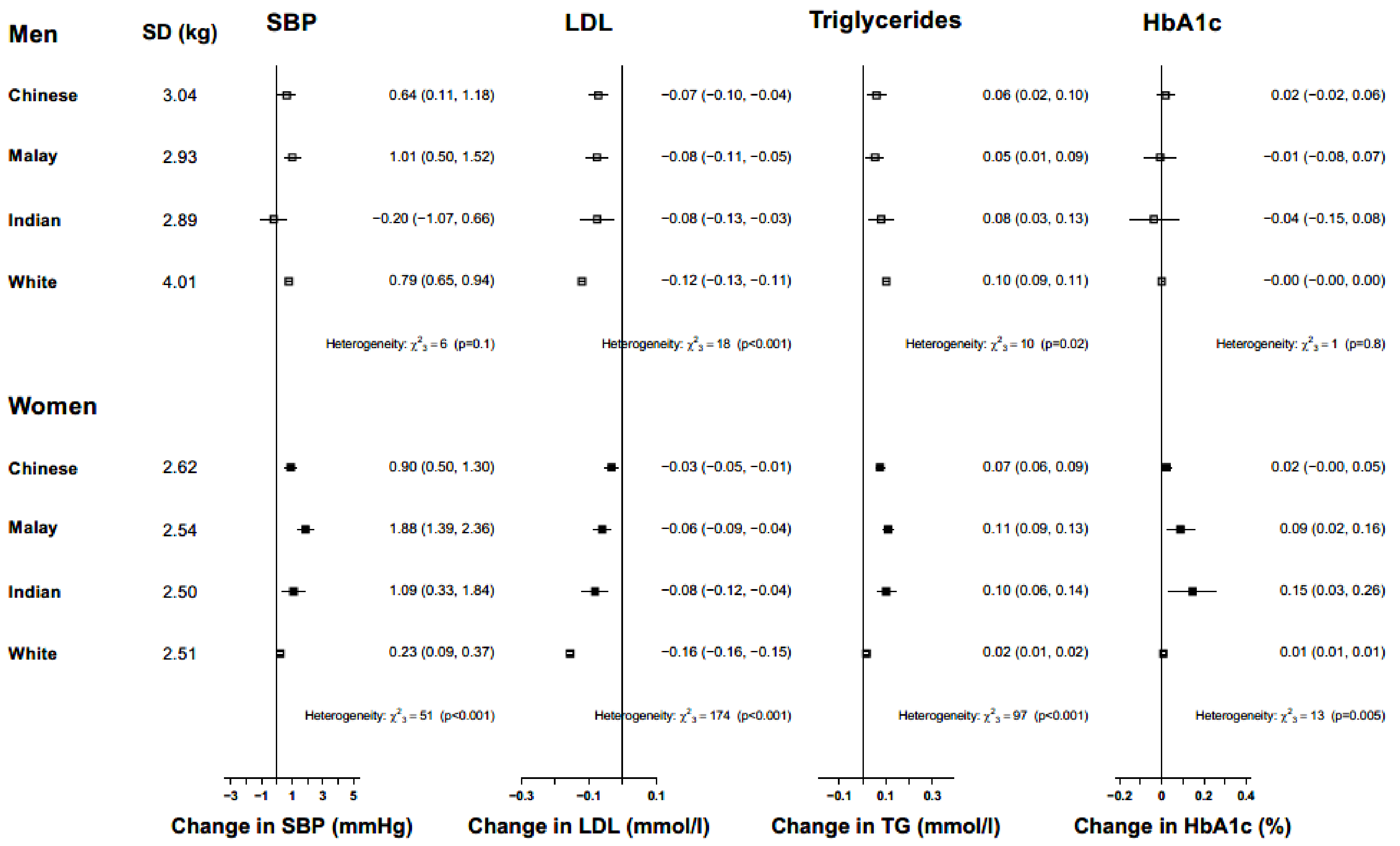

**Supplemental Figure e9. Fully adjusted associations between cardiovascular risk factors a 1 sex- and ethnic- specific SD change in <u>waist circumference</u>.**

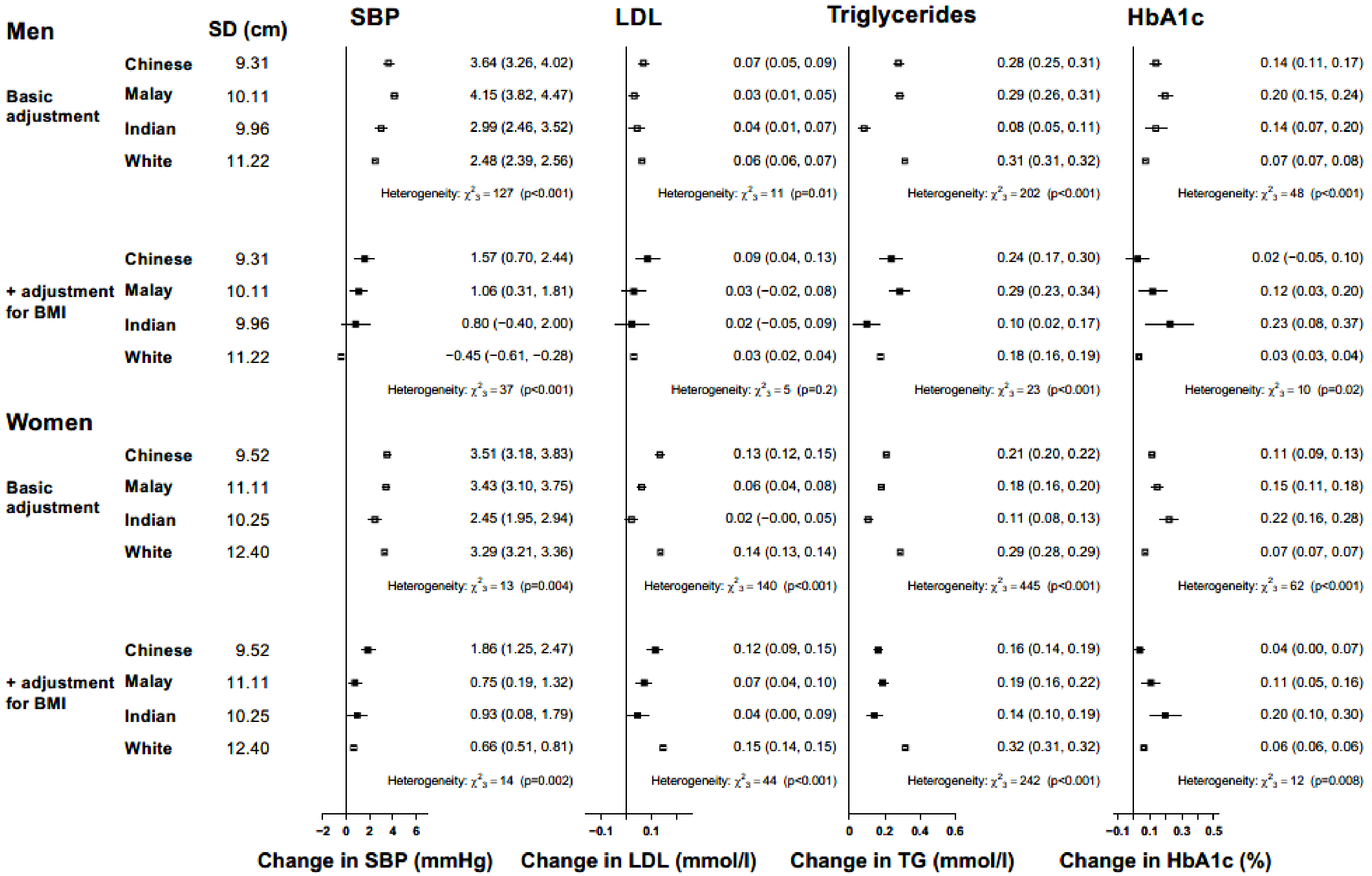

Supplement: Supplementary file 1 — Supplemental Material [file 41366_2023_1339_MOESM1_ESM.pdf]
